# Clinical management of patients with complicated multifocal disease

**DOI:** 10.1186/1470-7330-14-S1-O11

**Published:** 2014-10-09

**Authors:** Thomas Gruenberger

**Affiliations:** 1 Department of Surgery I, Rudolf Foundation Hospital, 1030 Vienna, Austria

## 

Deferent to the indication for surgical resection of liver metastases in previous years number, location and size are not the main targets to define resectability any more but the volume of the so called liver remnant, which has to have adequate inflow, outflow and biliary drainage. The magnitude of the remnant is dependent upon the function of the liver which nowadays is not only hampered by a potential underlying liver disease but very often damaged by previous extensive chemotherapy [1].

Liver surgery has become a safe procedure as a result of improved techniques, interdisciplinary management in radiologic assessment, oncological understanding, perioperative care and surgical specialisation.

When a patient is newly diagnosed with metastatic colorectal cancer (mCRC) the following issues should be discussed primarily in a multidisciplinary meeting: timing of metastatic diagnosis (metachronous/synchronous), primary insitu (yes/no; clinically symptomatic: y/n), affected organs, comorbidities, resectability status. Especially the last point has no general rules and is dependent upon the experience of the team and the specialised surgeon.

Initial resectability is categorized into three groups: resectable, potentially resectable (after downsizing through chemotherapy), unresectable [2]; the belonging of each patient to one of these groups can change within its course of disease and should therefore be rediscussed routinely every two months.

Every newly diagnosed mCRC patient should receive at least a short course of systemic chemotherapy (e.g. two months) to estimate his/her aggressiveness of disease and thereby define the right surgical candidate (the responding patient). Chemotherapy has become very effective with response rates (defined as complete or partial radiological response) in liver limited disease approaching 80% with very small percentage numbers of progressive disease (<5%) [3].

If a patient's disease has demonstrated to respond to chemotherapy every attempt should be made to perform resection with potential curative request in a fit patient who can tolerate major surgery. The technical options are various and range from a simple removal of half of the liver to the addition of resection of lesions in the remaining lobe or their destruction by thermal energy. This can be done after an increase of the initially too small remnant by using portal vein embolization (PVE) or by the use of a so called two stage procedure where one lobe is cleared of metastases at the first operation and the other lobe is resected secondarily after hypertrophy of the initially treated lobe (mostly with the addition of PVE). The resection itself is nowadays a bloodless procedure especially if sophisticated methods are used (e.g. CUSA technique) with the requirement of transfusions in less than 10% of patients in experienced centres [4].

Postoperative care is essential for the outcome of the patients and should include specialised anesthesiological surveillance and medical oncology advice after pathological work up of the resected metastases.

With this multidisciplinary approach to mCRC patients overall outcome has improved and reached median survival figures of 5 years [5].

## References

[B1] Krieger P-M Tamandl D Herberger B Evaluation of Chemotherapy-Associated Liver Injury in Patients with Colorectal Cancer Liver Metastases Using Indocyanine Green Clearance Testing Annals of Surgical Oncology 2011 18 1644 1650 10.1245/s10434-010-1494-1 21207168

[B2] Nordlinger B Van Cutsem E Gruenberger T Combination of surgery and chemotherapy and the role of targeted agents in the treatment of patients with colorectal liver metastases: recommendations from an expert panel Ann Oncol 2009 20 985 992 10.1093/annonc/mdn735 19153115

[B3] Masi G Loupakis F Salvatore L Bevacizumab with FOLFOXIRI (irinotecan, oxaliplatin, fluorouracil, and folinate) as first-line treatment for metastatic colorectal cancer: a phase 2 trial The Lancet Oncology 2010 11 845 852 10.1016/S1470-2045(10)70175-3 20702138

[B4] Tamandl D Gruenberger B Klinger M Liver Resection Remains a Safe Procedure After Neoadjuvant Chemotherapy Including Bevacizumab: A Case-Controlled Study Annals of Surgery 2010 252 124 130 10.1097/SLA.0b013e3181deb67f 20562613

[B5] Tomlinson JS Jarnagin WR DeMatteo RP Actual 10-Year Survival After Resection of Colorectal Liver Metastases Defines Cure Journal of Clinical Oncology 2007 25 4575 4580 10.1200/JCO.2007.11.0833 17925551

